# Analysis of a sample of type 2 diabetic patients with obesity or overweight and at cardiovascular risk: a cross sectional study in Spain

**DOI:** 10.1186/1756-0500-7-48

**Published:** 2014-01-21

**Authors:** María Orosia Lucha-López, Ana Carmen Lucha-López, Concepción Vidal-Peracho, José Miguel Tricás-Moreno, Elena Estébanez-De Miguel, Carlos Salavera-Bordás, Cesar Hidalgo-García

**Affiliations:** 1 Physiotherapy Research Unit, Faculty of Health Sciences - University of Zaragoza, C/Domingo Miral s/n, 50009, Zaragoza, Spain; 2 Physiotherapy Research Unit, Specialty Medical Center Grande Covian, SALUD, Avda Alcalde Caballero, 196, 50014, Zaragoza, Spain

**Keywords:** Diabetes Mellitus, Obesity, Cardiovascular risk, Genetics, Life habits, Health education, Physiotherapy

## Abstract

**Background:**

The multifactorial control of diabetes relies on interventions that provide patients with the best knowledge and resources available. The objective of this research was to analyze the clinical characteristics of a sample of people with type 2 diabetes at high cardiovascular risk, and establish possible links between disease control, family history and lifestyle, to improve the quality of interventions. Family history, lifestyle habits, blood pressure, anthropometric data and laboratory tests were analyzed in this descriptive and comparative cross-sectional study.

**Results:**

All patients had a pathological body mass index (BMI), and in those patients with a family history of diabetes, the disease was more serious and onset was earlier. Overall, 70.9% were taking drugs for arterial blood pressure management, with mean values within recommended limits; 87.1% were taking antihyperlipidemic drugs and had mean values for blood lipids within reference range; 93.5% were receiving oral antidiabetic drugs and/or insulin and had blood glucose and glycosylated hemoglobin (HbA_1c_) values higher than recommended limit; and 87% were taking antiplatelet drugs and had fibrinogen and ultrasensitive C-reactive protein higher than the normal range. High HbA_1c_ values were found in a high proportion of our sample who were not following a tailored diet (84.2%), and better BMIs were associated with moderate physical activity. Coexistence of somatic disorders (97.4% of the sample with musculoskeletal diseases) could lead to the lack of physical activity.

**Conclusions:**

This sample of patients with type 2 diabetes and at high cardiovascular risk, had acceptable metabolic control, facilitated by drug therapy. Family history of diabetes was associated with earlier disease onset and worse disease progression. Patients who were not following a tailored diet had worse HbA_1c_ values compared with those who were. Individuals who practiced moderate physical activity in line with international recommendations for weight maintenance had the best BMI values, but the high prevalence of comorbidities could adversely affect exercise habits. Appropriate use of medication, dietary advice, and tailored physiotherapy physical activity suitable for people with comorbidities should be included in multifactorial treatment strategies for these patients, particularly in the presence of a family history of diabetes.

## Background

Diabetes mellitus is currently a major public health problem [1]. In 2011, 366 million people worldwide were estimated to have diabetes. In Europe in 2011, 52.8 million people between 20 and 79 years of age had diabetes, with a prevalence of 8.1% [2]. In Spain between 2004 and 2010, 13.8% of people aged 18 and over had diabetes, similar to 2004–2006 rates, with a high rate (43%) of undiagnosed diabetes [3]. In Europe, one in 10 deaths in those aged between 20 and 79 years can be attributed to diabetes, with the vast majority of cases occurring in people over 50 years of age [1]. The causes of this high prevalence are attributable to both lifestyle and genetic factors. In terms of genetic factors, at least 40 loci associated with diabetes have been identified [4] and it has been reported that approximately 40% of people with type 2 diabetes have at least one relative that also has the disorder [5].

Diet and exercise are the lifestyle habits with the greatest influence on diabetes. In low- and middle-income countries – areas with a higher prevalence of the disease – the nutritional quality of food may be low, food with a very high glycemic index is consumed [6], and very little physical activity is done, because of technological advances and the lack of time. Therefore, control of this disease is a global health challenge and great efforts have been made in all health sciences fields to intervene in the process and to try to reverse it.

Recent studies published in Spain about the treatment of diabetes and the principal associated cardiovascular risk factors show encouraging results. In 2007, Orozco and colleagues found below-baseline values for glycosylated hemoglobin (HbA_1c_) in 50.6% of the population, blood pressure (BP) in 7.8% and low-density lipoprotein (LDL)-cholesterol in 5.9% [7]. Data published by Franch and collaborators in 2010 show a higher prevalence of below-baseline levels of these variables (59%, 28.51%, and 40.7% for HbA_1c_, BP and LDL-cholesterol, respectively) [8]. Mengual and colleagues reported similar findings in research published in 2010 (54.8%, 29.6%, and 40.6% for HbA_1c_, BP and LDL-cholesterol, respectively) [9]. Taken together, these show positive results in the treatment of diabetes mellitus, although it is necessary to study larger populations of people with diabetes, in line with levels recommended by reference scientific societies.

Multifactorial control of cardiovascular risk factors is important in achieving positive results. In the Steno study [10], intensive treatment for all cardiovascular risk factors, consisting of intensive drug therapy (antioxidant vitamins and insulin, lipid-lowering, antihypertensive and antiplatelet treatments), a low fat diet (<30% fat), and exercise, over a period of 13 years was shown to reduce complications by 29% and mortality by 20%.

The complexity of this intensive and multifactorial control of diabetes [11] and the improved health and increased survival that can be achieved, highlight the importance of interventions that provide the patient with the best knowledge and resources available [12].

The objective of this research was to analyze the clinical characteristics of a sample of people with type 2 diabetes at high cardiovascular risk, and establish possible links between disease control, family history, and lifestyle, to improve the quality of interventions.

## Methods

### Study and sample selection

A descriptive and comparative cross-sectional study was developed, comprising a sample of 38 people with type 2 diabetes and a pathological body mass index (BMI) who attended a specialized Endocrinology and Nutrition outpatient clinic at the Hospital Royo Villanova (Grande Covian Specialty Medical Center) in Zaragoza, Spain.

Inclusion criteria were as follows: diagnosis of type 2 diabetes, according to the American Diabetes Association (ADA) 2012 criteria [13]; diabetes duration >10 years; BMI ≥25; and age >45 years.

The Spanish Society for the Study of Diabetes and Obesity (SEEDO) defines pathological BMI as follows: overweight grade I, 25.0–26.9 kg/m^2^; overweight grade II (pre-obesity), 27.0–29.9 kg/m^2^; obesity type I, 30.0–34.9 kg/m^2^; obesity type II, 35.0 to 39.9 kg/m^2^; obesity type III (morbid), 40.0–49.9 kg/m^2^; and obesity type IV (extreme) ≥50 kg/m^2^[14].

All patients who met the inclusion criteria and attended consultations were included. They voluntarily accepted to participate after receiving information on the study, so we used a convenience, consecutive, non-probabilistic sampling method. All participants signed an informed consent form and were told that they could leave the study at any time and for any reason. The Department of Physical Therapy and Nursing at the University of Zaragoza approved the study, which complied with the ethical requirements of the Declaration of Helsinki [15].

### Measurements

Medical histories for each patient were taken including sex, age, age at diabetes onset, family history [16], comorbidities, diet in the past year, physical activity, smoking status, and medication use.

The measurement of systolic blood pressure (SBP) and diastolic blood pressure (DBP) was performed using a sleeve adapted to obese patients [17] after a period of approximately 15 minutes in a sitting position, with one tube and a clock tensiometer (Riester, Jungingen, Germany).

Anthropometric data were obtained according to the protocol of the International Society for the Advancement of Kinanthropometry (ISAK) [18]. Height was measured with a wall stadiometer (removable stadiometer, scope 30–220 cm, Seca, Hamburg, Germany,), weight with an electronic scale (Tanita TBF 300, Tanita, Amsterdam, the Netherlands) and waist circumference with an inextensible tape (plastic tape, 0–2 m, Tecsymp Instruments, Barcelona, Spain).

The assessment of blood parameters was performed in the laboratory of Grande Covian Specialty Medical Center, by drawing blood after a fasting period of not less than 12 hours. Biochemical analyses took place at the Grande Covián laboratory. Total cholesterol, (HDL)-cholesterol, (LDL)-cholesterol, triglycerides and fibrinogen were determined by nephelometry (Dade Behring Corporation, Berlin, Germany). Fating blood glucose, HbA_1c_, and ultrasensitive C reactive protein (us-CRP) were determined using a selective modular analyzer (Roche, Basel, Switzerland). Accreditation National Entity (ENAC) validated the quality control of the laboratory (accreditation number: 742/LE1586).

### Statistical analysis

Data were analyzed with SPSS version 16.0 (SPSS Inc., Chicago, USA). Descriptive statistics were calculated (the mean and standard deviation (SD) and 95% confidence interval (CI) for quantitative variables and percentages for qualitative variables).

We evaluated the magnitude of the association between quantitative variables with Spearman’s correlation coefficient (rho).

The Mann–Whitney U test was used for the comparison of independent variables, which included family history of diabetes (yes/no), dietary habits (no diet/diet adapted to the clinical situation), and physical activity (no physical activity/moderate physical activity). The power of these tests was calculated with the effect size detected in the study. A level of significance of 5% (p <0.05) was established to reject the null hypothesis.

## Results

General characteristics of the sample are shown in Table 1.

**Table 1:** General sample characteristics

**General characteristics of the sample**	**Percentage or mean ± SD [95% CI]**
Females	74% (n = 28)
Males	26% (n = 10)
Age (years)	59.1 ± 7.6 [56.6-61.6]
Age at the onset of diabetes (years)	48.7 ± 10.2 [45.4-52.1]
Family history of diabetes	70% (n = 26)
Adapted diet in the last 12 months	15.8% (n = 6)
Smoking habit	0% (n = 0)

When stratified according to family history of diabetes, there were significant differences in age of diabetes onset, weight, waist circumference, total cholesterol, and fasting blood glucose (Mann–Whitney U test). See Table 2.

**Table 2:** Comparison according to family history of diabetes

**Variables with differences according to family history of diabetes**	**With family history of diabetes (n = 26)**	**Without family history of diabetes (n = 12)**	**p value**
	**Mean ± SD/power value**	**Mean ± SD/power value**	
Age at the onset of diabetes	45 ± 7.9/0.9	56.3 ± 10.8/0.7	0.002
Weight	89.6 ± 13.5/0.9	77.9 ± 14/0.5	0.019
Waist circumference	110.1 ± 11.4/0.9	99.9 ± 11.2/0.6	0.026
Total cholesterol	176.3 ± 19.9/0.8	160.2 ± 28.6/0.3	0.031
Glucose	149.3 ± 42.6/0.7	120.2 ± 27.1/0.7	0.045

Descriptive data on cardiovascular risk components are shown in Table 3 and drug prescriptions in Table 4. Of the study sample, 93.5% were taking drugs to control blood glucose (41.9% were treated with oral agents, 6.4% with insulin and 45.2% with oral agents associated with insulin).

**Table 3:** Cardiovascular risk parameters

**Cardiovascular risk components**	**Values in the sample mean ± SD [95% CI]**	**Reference values**	**Percentage values of the sample within reference**
BMI	34.8 ± 5.8 [32.9-36.7]	18.5-24.9^1^	0% (n = 0)
Weight (kg)	85.5 ± 14.9 [80.6-90.4]		
Waist circumference (cm)	106.7 ± 12.2 [102.7-110.7]	< 88 females/102 males^2^	5.3% (n = 2) females/13.2% (n = 5) males
SBP (mmHg)	133.6 ± 13.8 [128.4-138.9]	< 140^1^	47.4% (n = 18)
DBP (mmHg)	79.3 ± 5.9 [7781.–6]	< 90^1^	68.4% (n = 26)
Glucose (mg/dl)	141.3 ± 40.7 [127.5-155]	< 100^3^	11.1% (n = 4)
Total cholesterol (mg/dl)	171.9 ± 23.4 [164180]–	< 200^1^	91.7% (n = 33)
HDL-c (mg/dl)	56.8 ± 11 [5360.–5]	> 50^1^	66.7% (n = 24)
LDL-c (mg/dl)	92.1 ± 20.7 [85.2-99.1]	< 130^1^	89.5% (n = 34)
Triglycerides (mg/dl)	123.1 ± 76.7 [97.2-149]	< 200^1^	91.7% (n = 33)
HbA_1c_ (%)	6.8 ± 1.3 [6.3-7.2]	< 7%^3^	44.7% (n = 17)
Fibrinogen (mg/dl)	339.3 ± 85.8 [309.8-368.8]	200-400^4^	77.1% (n = 27)
us-CRP (mg/L)	6.3 ± 7.4 [3.8-8.8]	< 3^1^	44.7% (n = 17)

^1^SEEDO 2007, ^2^ATP III 2002, ^3^ADA 2012, ^4^Gailani D, Neff AT 2008.

ADA = American Diabetes Association; ATPIII: adult treatment panel III; BMI = body mass index; HbA_1c_ = glycosylated hemoglobin; HDL-c = high-density lipoprotein cholesterol; LDL-c = low-density lipoprotein cholesterol; SEEDO = Spanish Society for the Study of Diabetes and Obesity; us-CRP = ultrasensitive C reactive protein.

**Table 4:** Medications used

**Type of drug**	**Percentage of the sample**
Drugs to control blood pressure	70.9% (n = 27)
Hypolipidemic drugs	87.1% (n = 33)
Oral antidiabetic and/or insulin	93.5% (n = 36)
Antiplatelet drugs	87% (n = 33)

Only 15.8% of people were on a diet tailored to their clinical condition (Table 1). When comparing this group with the group that did not diet, differences in HbA_1c_ values were observed (Mann–Whitney U test). See Table 5.

**Table 5:** Comparison according to having followed or not followed a tailored diet during the last year

**Variables with differences according to diet**	**Diet (n = 6)**	**No diet (n = 32)**	**p value**
	**Mean ± SD/power value**	**Mean ± SD/power value**	
HbA_1c_	5.85 ± 0.87/0.5	6.96 ± 1.31/0.9	0.037

HbA_1c_ = glycosylated hemoglobin.

**Figure 1:**
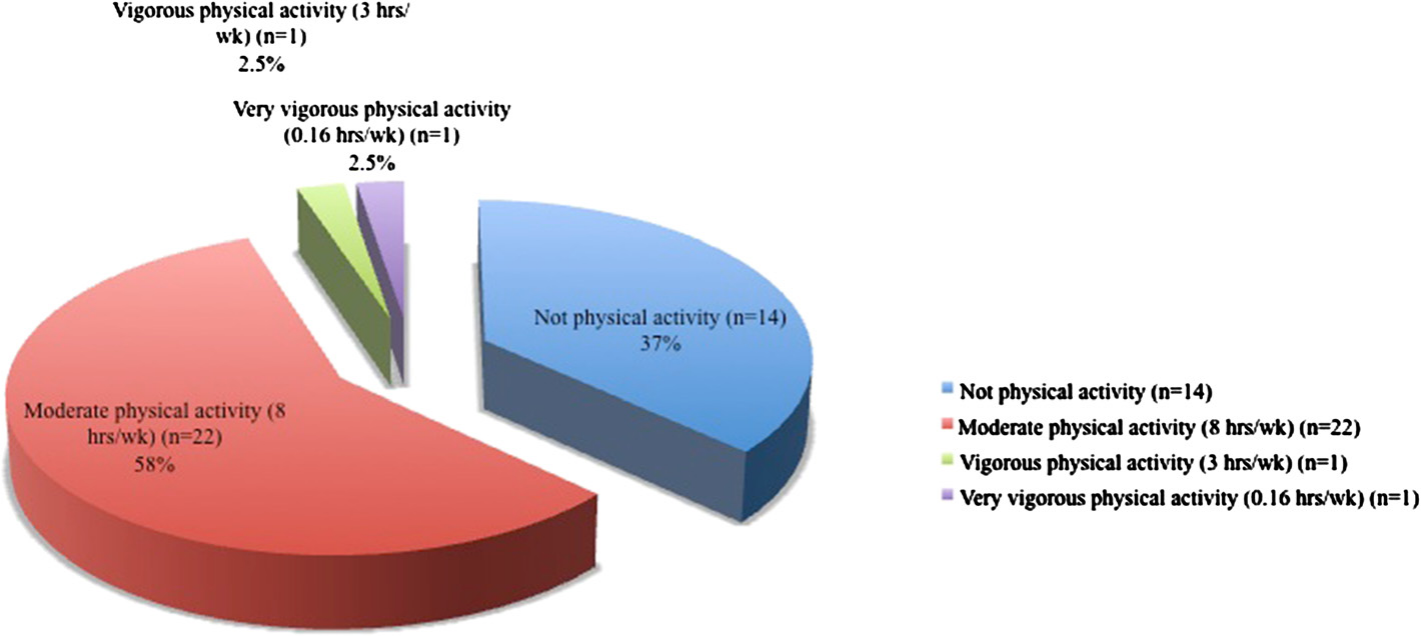
Dedication to physical activity.

The type of physical activity undertaken is shown in Figure 1. There was a statistically significant difference in BMI between individuals who undertook moderate physical activity compared with those who did not (p = 0.032) (Table 6).

**Table 6:** Comparison according to the undertaking of physical activity

**Variables with differences according to dedication to physical activity**	**Not physical activity (n = 14)**	**Moderate physical activity (8 h/sem) (n = 22)**	**P value**
	**Mean ± SD/power value**	**Mean ± SD/power value**	
BMI	37.7 ± 5.8/0.4	33.7 ± 5.2/0.7	0.032

BMI = body mass index.

**Figure 2:**
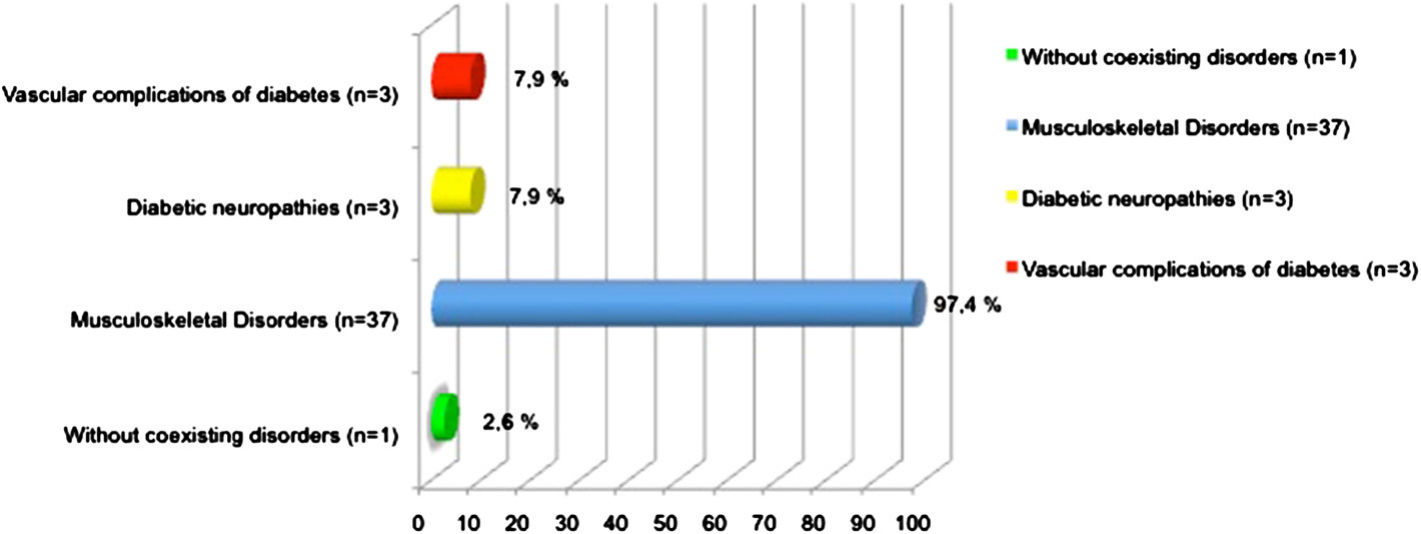
Other somatic disorders.

The percentages of patients with coexisting somatic disorders and sources of disability are shown in Figure 2.

Table 7 shows the correlation between various clinical variables.

**Table 7:** Correlation between clinical variables

**Clinical variables**	**Glucose**	**Total cholesterol**	**LDL-c**	**Triglycerides**	**us-CRP**
Age at the onset of diabetes	(-) 0.375*				
BMI		0.407*		0.397*	0.345*
Weight	0.427**	0.528**	0.350*	0.464**	0.395*
Waist circumference	0.371*		0.413*	0.444**	0.401*
Triglycerides	0.375*				0.368*

**Correlation is significant at the 0.01 level (bilateral). *Correlation is significant at the 0.05 level (bilateral).

BMI = body mass index; LDL-c = low-density lipoprotein cholesterol; us-CRP = ultrasensitive C reactive protein.

## Discussion

This study included 38 people aged over 45 years with type 2 diabetes of >10 years duration. Almost three-quarters of the sample were female, and 70% had a family history of diabetes. This proportion was slightly higher than that outlined by previous studies provide data on approximately 40% [5]. The age of onset of diabetes in our study was greater by approximately 11 years in patients with a family history of diabetes compared with the patients without a family history of diabetes. A family history of diabetes negatively influenced weight, waist circumference, blood glucose levels and cholesterol levels. These results suggest the importance of the genetic component in the pathogenesis of this process [19,20] although it is known that environmental factors also play an important role [21].

The mean BMI in our study was 34.81 kg/m^2^, and fat distribution was central, with a high percentage of the sample having a waist circumference higher than reference values [22]. This type of adiposity is more frequently associated with increased cardiovascular risk, dyslipidemia and insulin resistance [23]. In this study, people with larger waist circumference had higher blood glucose, triglycerides, LDL cholesterol and us-CRP, supporting the importance of the loss of fat mass to facilitate the treatment of type 2 diabetes [24].

Regarding concomitant drug use, 70.9% of our patients were receiving drugs to control blood pressure, and 47.4% and 68.4% of study participants had SB, and DBP, respectively, within the normal range. [14]. The positive effects of blood pressure control in the course of the disease may have prevented the development of diabetic nephropathy, as none of the patients showed this complication, despite having been diagnosed with diabetes for a long period of time [25].

A total of 87.1% of the sample was receiving lipid-lowering therapy, and 91.7%, 66.7%, 89.5% and 91.7% of patients had total cholesterol, HDL-cholesterol, LDL-cholesterol and triglycerides below the recommended levels [26].

By contrast, despite therapy with oral antidiabetic drugs and/or insulin, in 93.5% of patients, the mean blood glucose level was above target values. However, 44.7% had HbA_1c_ below the target value of 7% (53 mmol/mol) [13,27]. Eighty-seven per cent of the sample were receiving antiplatelet drug treatment, achieving a mean fibrinogen value of 339.3 mg/dL, with 77.1% of the sample having values below baseline [28].

Analysis of fibrinogen as a sensitive indicator of inflammation [29] can be complemented with the analysis of the us-CRP, for which values in our study were higher than reference values [25], and only 44.7% of subjects were within the recommended levels. This observed pro-inflammatory state might be related to insulin resistance, because it has been demonstrated that the plasma us-CRP might be a marker of risk for the development of macrovascular disease and diabetes mellitus, even independently of obesity [30].

These data reflect that patients had acceptable metabolic control according to ADA [31] and International Diabetes Federation (IDF) [32] criteria, although the pro-inflammatory markers indicate that this sample was at high cardiovascular risk. The current clinical evidence and consensus recommendations support that type 2 diabetes is associated with high cardiovascular risk, particularly when other risk factors are present, and at 10 years after diagnosis [22,30].

High HbA_1c_ values were found in a high proportion of our sample who were not following a tailored diet (84.2%) [33], in agreement with recent studies that continue to show the benefits of the adjustments in diet to control glucose metabolism [34-36].

Another key aspect of the treatment of diabetes is physical activity [37]. A recent study has shown that higher levels of physical activity are associated with decreased risk of developing diabetes, even independently of the general and abdominal fat percentage [38]. In our study, 57% of patients reported undertaking moderate physical activity, at an average duration of 8 hours per week, which is sufficient for the maintenance of weight loss in obese people according to international recommendations [39]. However, up to 37% reported undertaking no physical activity, and when comparing the two groups, the most favorable BMI values were observed in the physically active group, as has been reported by others [40].

One barrier to patients with diabetes undertaking physical activity is the high prevalence of coexisting conditions that may limit their functional ability and cause alterations in sensitivity [41]. In the United States, 30% of people with type 2 diabetes have impaired sensation in the feet, and over 50% have osteoarthritis [42]. In our sample, up to 97.4% had musculoskeletal disorders, including osteoarthritis, 7.9% had diabetic neuropathy and 7.9% had vascular complications, which may have decreased adherence to physical activity because of pain and decreased function. To facilitate exercise in patients with such problems, exercise must be adapted to the clinical situation and, ideally, treated specifically. Reinforcing this, a recent study has demonstrated the effectiveness of a physiotherapy exercise program for patients with diabetes, with good results in controlling pain and improving mobility [43].

A limitation to our study is that patients joined the study voluntarily, leading to the possibility of non-response bias on the part of the population that would not, or could not, be included. The strict inclusion criteria of the study prevented the recruitment of a larger sample size, which would have improved the possibilities to estimate the true population values, and larger studies in similar populations are needed to confirm our findings.

## Conclusions

This sample of patients, at high cardiovascular risk and with a strong influence of a family history of diabetes, had acceptable metabolic control, facilitated by intensive drug therapy. Family history of diabetes was associated with earlier onset and worse disease progression. Patients who were not following a tailored diet had worse HbA_1c_ values compared with those who were. The proportion of the sample that practiced moderate physical activity in line with international recommendations for weight maintenance had the best BMI values, but the high prevalence of comorbidities could adversely affect exercise habits. Appropriate use of medication, dietary advice, and tailored physiotherapy physical activity suitable for people with comorbidities should be included in multifactorial treatment strategies for these patients, particularly in the presence of a family history of diabetes.

### Availability of supporting data

The data sets supporting the results of this article are included within the article.

## Abbreviations

ADA: American Diabetes Association; ATPIII: Adult Treatment Panel III; BMI: Body mass index; BP: Blood pressure; CI: Confidence interval; Cm: Centimeter; DBP: Diastolic blood pressure; dL: Deciliter; DNA: Deoxyribonucleic acid; ENAC: Accreditation National Entity; HbA1c: Glycosylated hemoglobin; HDL: High-density lipoprotein; hrs/wk: Hours/week; IDF: International Diabetes Federation; ISAK: International Society for the Advancement of Kinanthropometry; Kg: Kilogram; L: Liter; LDL: Low-density lipoprotein; Mg: Milligram; mmHg: Millimeter of mercury; SBP: Systolic blood pressure; SD: Standard deviation; SEEDO: Spanish Society for the Study of Diabetes and Obesity; us-CRP: ultrasensitive C reactive protein.

## Competing interests

The authors declare that they have no competing interests.

## Authors’ contributions

JMTM and CVP contributed to the conception and design of the work. MOLL, ACLL, EED and CSB organized the sample collection and data preparation, performed data collection, analysis and interpretation. CHG prepared drafts of the article. MOLL, ACLL, CVP, JMTM, EED, CSB and CHG critically reviewed its comprehensive content and finally approved the version to be submitted for publication.
